# Modulation of large dense core vesicle insulin content mediates rhythmic hormone release from pancreatic beta cells over the 24h cycle

**DOI:** 10.1371/journal.pone.0193882

**Published:** 2018-03-15

**Authors:** Aurore Quinault, Corinne Leloup, Geoffrey Denwood, Coralie Spiegelhalter, Marianne Rodriguez, Philippe Lefebvre, Nadia Messaddeq, Quan Zhang, Catherine Dacquet, Luc Pénicaud, Stephan C. Collins

**Affiliations:** 1 CSGA, AgroSup Dijon, Centre National de la Recherche Scientifique, Institut National de la Recherche Agronomique, Université de Bourgogne Franche-Comté, 9E Boulevard Jeanne d'Arc, Dijon, France; 2 Oxford Centre for Diabetes, Endocrinology and Metabolism, Churchill Hospital, University of Oxford, Headington, Oxford, United Kingdom; 3 IGBMC, 1 Rue Laurent Fries, Illkirch-Graffenstaden, France; 4 Metabolism Discovery Research Pole of Therapeutical innovation Institut de Recherche Servier, 11 rue des Moulineaux Suresnes, France; 5 European Genomic Institute for Diabetes and UMR 1011 Inserm Université Nord de France-Institut Pasteur de Lille, Boulevard du Professeur Leclerc, Lille, France; 6 Biotechnology and Biomarker Research, Institut de Recherche Servier, 125 Chemin de Ronde, Croissy sur Seine, France; McGill University, CANADA

## Abstract

The rhythmic nature of insulin secretion over the 24h cycle in pancreatic islets has been mostly investigated using transcriptomics studies showing that modulation of insulin secretion over this cycle is achieved via distal stages of insulin secretion. We set out to measure β-cell exocytosis using in depth cell physiology techniques at several time points. In agreement with the activity and feeding pattern of nocturnal rodents, we find that C57/Bl6J islets in culture for 24h exhibit higher insulin secretion during the corresponding dark phase than in the light phase (Zeitgeber Time ZT20 and ZT8, respectively, *in vivo*). Glucose-induced insulin secretion is increased by 21% despite normal intracellular Ca^2+^ transients and depolarization-evoked exocytosis, as measured by whole-cell capacitance measurements. This paradox is explained by a 1.37-fold increase in beta cell insulin content. Ultramorphological analyses show that vesicle size and density are unaltered, demonstrating that intravesicular insulin content per granule is modulated over the 24h cycle. Proinsulin levels did not change between ZT8 and ZT20. Islet glucagon content was inversely proportional to insulin content indicating that this unique feature is likely to support a physiological role. Microarray data identified the differential expression of 301 transcripts, of which 26 are miRNAs and 54 are known genes (including *C2cd4b*, a gene previously involved in insulin processing, and clock genes such as *Bmal1* and *Rev-erbα*). Mouse β-cell secretion over the full course of the 24h cycle may rely on several distinct cellular functions but late night increase in insulin secretion depends solely on granule insulin content.

## Introduction

Links between circadian dysfunction and Diabetes has been evidenced in several models where clock genes were either mutated to knocked out [1, 2]. A complex picture of circadian gene expression in pancreatic islets has emerged, some KO models showing impairment of β-cell mass [2, 3] and distal stages of insulin secretion as the underlying pathophysiological mechanisms. However, direct functional evidence remains scarce. Recently, new mechanisms controlling the beta cell output across the 24h cycle was unraveled using *in vivo* and functional assays. One study reported that ATP production via modification of *Ucp2* expression modifies insulin secretion [4] although islet insulin content was not measured. In contrast, others have established that the insulin genes display circadian expression and may underly the day/night rhythmicity of Glucose Stimulated Insulin Secretion (GSIS) [5]. Which cellular processes are at play in the unaltered β-cell during the 24 hour cycle thus remains an open question, and measures of metabolism and exocytosis that do not rely on measures of insulin content are needed.

Here, we show that in the absence of exocytosis or metabolic upregulation, mouse β-cells are programmed to increase insulin content per granule during late night.

## Material and methods

### Animal husbandry

Experiments were performed in agreement with the European Directive 2010/63/UE and approved by the French Ministry of Research and the local ethic committee of the University of Burgundy (C2EA Grand Campus Dijon N° 105). 10-week-old *C57Bl/6J* (Charles River) mice were housed on a 12h light/dark cycle with the light period starting either at 7:00 or at 19:00. In both cases, the time of lights on defines Zeitgeber (ZT) 0. Mice had free access to food and water at all times. After a 4-weeks acclimatization period, mice were killed at 15:00 by cervical dislocation under light (ZT8 and ZT12) or dark conditions (ZT20) and islets isolated by collagenase digestion and handpicking, followed by culture for 24h as described previously [6]. All further experiments unless otherwise stated were performed at 15:00 or on a 4h window around that time (for electrophysiological and fluorimetric experiments). The normal-cycle mouse islet group is subsequently referred to as the ZT8 group, whilst the inverted cycle mouse islets are referred to as ZT20. An additional time point was investigated (ZT12) for exocytosis. To this effect, mice kept on a normal cycle were killed at 15:00 (ZT8 independent control) and 19:00 (ZT12), and islets were cultured for 24h before experiments.

### Measurement of hormone secretion

Islets were cultured in RPMI 1640 together with 11mM G based on recommended publications [7, 8]. Static measurements of insulin secretion were performed in 96 well plate format plates using an extracellular medium containing (in mM): 120 NaCl, 4.7 KCl, 25 NaHCO_3_, 1.2 KH_2_PO_4_, 1.2 MgSO_4_, 10 HEPES (pH 7.4 with NaOH), 2.5 CaCl_2_, 0.5 mg/ml BSA free fatty acid free. Islets were kept one hour at 5 mM G and the supernatant was replaced with test solutions containing either 2.8 or 16.7 mM G for another hour. The supernatant was collected and to determine total content, hormones were extracted from pellets using 95:5 ethanol:acetic acid. Insulin, proinsulin and glucagon were measured using commercial ELISA kits (Alpco, Mercodia and Millipore, respectively) on both supernatant and pellet.

### Measurement of DNA content

Total DNA content was measured on islets at ZT8 and ZT20 using Qubit dsDNA-HS assay kit (ThermoFisher) according to the manufacturer’s instructions.

### Intracellular Ca^2+^ measurements

Intracellular calcium concentration ([Ca^2+^]_i_) was assessed on islets using a Till photonics system fitted on an upright inverted IX 70 Olympus microscope, allowing ratiometric measurements with fura-2AM (Molecular Probes) as described previously [9].

### Whole-cell measurements of Ca^2+^ currents and exocytosis

For patch-clamp measurements, islets were dissociated into single β-cells. Insulin-secreting β-cells were identified based on their larger size reflected by their higher capacitance (>5.5 pF) and complete inactivation of the Na^+^ current at -70 mV [10]. Whole-cell Ca^2+^ currents (using Ba^2+^ as the charge carrier to prevent Ca^2+^-mediated inactivation of the channels and to increase the magnitude of the current) and exocytosis (Ca^2+^ present in the extracellular solution) were recorded using an EPC-10 amplifier and the Pulse software (Heka Electronics, Lamprecht/Pfalz, Germany) as described previously [11].

### Electron microscopy

Groups of 50 isolated islets were fixed in 2.5% glutaraldehyde and 2.5% paraformaldehyde in cacodylate buffer (0.1 M, pH 7.4) and washed in cacodylate buffer for further 30 minutes. Samples were postfixed in 1% osmium tetroxide in 0.1M cacodylate buffer for 1 hour at 4°C and stained with 2% uranyl acetate for 1h at 4°C. The samples were dehydrated through graded alcohol (50, 70, 90, and 100%) and propylene oxide. Samples were embedded in Epon 812. Ultrathin sections were cut at 70nm and contrasted with uranyl acetate and lead citrate and examined at 70kV with a Morgagni 268D electron microscope. Images were captured digitally by Mega View III camera (Soft Imaging System) and analysed using ImageJ.

### qRT-PCR and microarray studies

Groups of 20 islets were washed by centrifugation in cold PBS (Ozyme, Saint-Quentin-en-Yvelines) and immediately homogenized in lysis solution (RLT buffer provided by mRNA extraction kit), frozen and stored at -80°C until mRNA extraction. Total islet mRNA was extracted using the RNeasy Micro kit (Qiagen, Courtabœuf) according to the manufacturer’s protocol. Quantity and quality (RNA quality indicator; RQI) of total RNA were checked through the Experion automated electrophoresis system (Bio-Rad, Marne la coquette) and software. Samples with a RQI above 7 were accepted. cDNAs were synthesized using the QuantiTect Reverse Transcription Kit (Qiagen, Courtabœuf). Reverse transcription were performed in triplicate. Real time RT-PCR was performed with pre-designed Taqman Gene expression assays (Life Technologies, Saint Aubin). Primers used for qRT-PCR are detailed in S1 Table and listed here: *Rev-erb α* (alias Nr1d1; Mm00520708_m1), *Rev-erb β* (alias Nr1d2; Mm00441730_m1), *Bmal1* (alias Arntl; Mm00500226_m1); *Ins1* (Mm01950294_s1); *Ins2* (Mm00731595_gH); *Glucagon* (Mm01269055_m1) and *36B4* (alias Rplp0; Mm00725448_s1). The 20μl PCR reactions contained 2μl cDNA, 10μl Taqman Fast Advanced Master Mix (Life technologies, Saint Aubain), 1μl Taqman Gene expression Assay and 7μl H_2_O. Samples were analyzed on the StepOnePlus Real-Time PCR system (Applied Biosystem, Saint Aubin). Cycling parameters for real-time RT-PCR were as follows: 50°C for 2 minutes, 95°C for 10 min followed by 45 cycles of 95°C for 15 seconds and 60°C for 1 minute. Relative expression levels were determined by StepOne software using comparative CT method to normalize target gene mRNA to *36B4* as published before [12].

In a separate group of experiments, samples with a RQI above 7.5 were used for microarray analysis. Labeled and fragmented cRNA was hybridized to the Affymetrix 430 2.0 whole mouse genome microarray and processed on an Affymetrix GeneChip Fluidics Station 450 and Scanner 3000 (Affymetrix).

### Statistical analysis

Statistical analysis was performed using two-tailed t-test, ANOVA repeated measures or using a Mann-Whitney U-test (ultrastructural data) where appropriate using the statistical package “R”.

## Results

### Validation of experimental model via rhythmic *Rev-erb α* and *Bmal1* gene expression profiles

**Fig 1:**
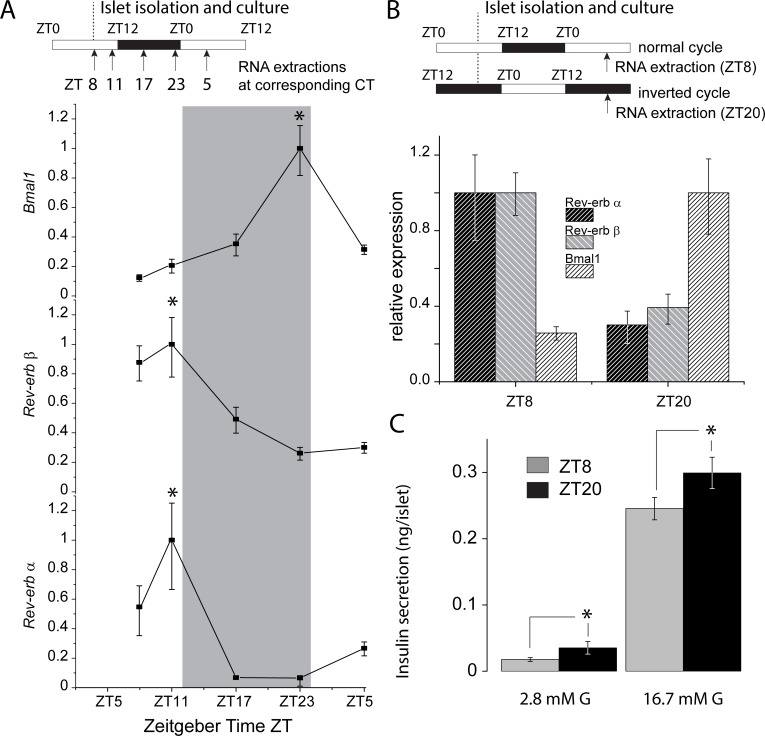
Expression levels of main circadian genes and corresponding Glucose Stimulated Insulin Secretion (GSIS) at peaks of expression. *A*: 24h expression profile of *rev-erb α* and β and *Bmal1* in islets cultured for 24h and sampled every 6h (except first two sample times collected at 15:00 (ZT8) and 19:00 (ZT11)). *B*: Expression profiles at ZT8 and ZT20 of *rev-erb α*, *rev-erb* β, and *Bmal1* in islets from normal or inversed cycle mice after 24h of culture. Islet isolation and sampling time are indicated in schematics. Data are from 4 animals for (*A*) and 3 animals for (B), with 2 technical replicates per animal and per ZT where applicable. White circles = Rev-erb *α*; White squares = Rev-erb β; Dark squares = Bmal1; *P<0.05 by ANOVA (A). *C*: Basal (2.8 mM glucose) and Glucose induced (16.7 mM glucose) insulin secretion from ZT8 and ZT20 islets. Grey bars = ZT8; Dark bars = ZT20; N = 20–60 experimental replicates across 6 animals per ZT. *P<0.05 using a *t-test*.

The circadian system of pancreatic islets is totally preserved in the first few days of culture without need for a synchronization pulse [13]. We verified and confirmed the cyclic expression of Rev-Erb α and β (*Nr1d1* and *Nr1d2*) and Bmal1 (*Arntl*) by qRT-PCR on islets cultured for 24h and sampled every 6h (schematics in Fig 1A). The two *Rev-erb* isoforms peaked at ZT8 whilst *Bmal1* peaked at ZT20 as previously established [5] (Fig 1A). We subsequently focused on time points with maximal and minimal relative expression of *Rev-erb α*, ZT8 and ZT20, respectively. Since islet culture duration has a significant effect on β-cell Ca^2+^-channel properties (Figures A and B in S1 Fig), mice were housed in normal or inversed cycle conditions as shown in Fig 1B schematics to standardize culture length (24h). Relative expression of the aforementioned circadian genes (Fig 1B) followed the same pattern as in Fig 1A, consistent with an entrainment of the clock in the islets under both light/dark cycles, and validating our experimental paradigm.

### Increased insulin secretion at ZT20 is not due to [Ca^2+^]_i_, Ca^2+^ channel properties and exocytosis

**Fig 2:**
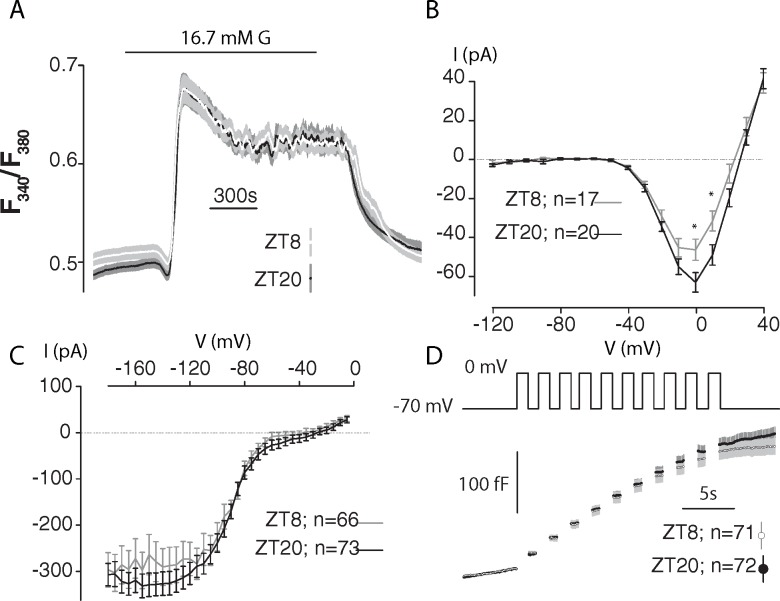
No change in [Ca^2+^]_i_ signaling, Na^+^, Ca^2+^ currents and exocytosis (measured via whole cell capacitance) between ZT8 and ZT20. *A*: Glucose-induced [Ca^2+^]_i_ transients in ZT8 or ZT20 islets. The glucose concentration was varied as indicated above the traces from a basal concentration of 2.8 mM glucose. Glucose-induced oscillations during the plateau phase were 0.085±0.0081 and 0.076±0.0054 in amplitude (Ratio of F_340_/F_380_), whilst oscillation period was 111±11 s and 92±7 s in ZT8 and ZT20 islets, respectively. n = 41 and n = 52 islets across a minimum of 6 animals for ZT8 and ZT20, respectively. *B*: Peak current (*I*)-voltage (*V*) relationship for voltage-gated Ca^2+^ currents recorded from single ZT8 or ZT20 β-cells using Ba^2+^ in the extracellular solution as a surrogate to Ca^2+^ during depolarizations from -70 mV to membrane potentials between -60 and +80 mV (n = 17 and 20 β-cells from 3 mice for ZT8 and ZT20, respectively). * p<0.05 using a t-test. The horizontal dotted line indicates zero current level. *C*: Na^+^ currents from single ZT8, n = 66 or ZT20, n = 73 β-cells across 3 mice per ZT. *D*: Increase in membrane capacitance (Δ*C*) during a train of ten 500 ms depolarizations from -70 mV to 0 mV (*V*) in single ZT8, n = 71 or ZT20, n = 72 β-cells across 3 mice per ZT. Traces are mean ± S.E.M. Grey lines = ZT8; Dark lines = ZT20 on all subpanels.

Both basal and GSIS were upregulated during the corresponding mouse awake/activity phase (Fig 1C). Basal insulin release was increased by 100% and GSIS by 21% (p<0.05). We measured β-cell intracellular Ca^2+^ concentration ([Ca^2+^]_i_) in cultured islets from normal and inverted cycle mice in response to glucose (Fig 2A). Both islet sources responded to 16.7mM glucose, with similar increases in [Ca^2+^]_i_ and fast oscillation patterns. The magnitude and gating of the Ca^2+^ currents were identical at ZT8 and ZT20 in the physiological range of β-cell electrical activity (-70 to -10 mV) (Fig 2B). Unlike mouse β-cells, recent studies on human islets have highlighted that Na^+^ channels participate in insulin secretion [10]. We characterized Na^+^ currents but found no difference between the two groups (Fig 2C). Cell membrane capacitance was not different at ZT8 and ZT20 before the depolarization train (6.94±0.2 and 7.17±0.3 pF at ZT8 and ZT20, respectively p = 0.52). The total increase in cell membrane capacitance elicited by the train was ~130 fF in both ZT8 and ZT20 (Fig 2D).

### Higher intragranular content correlates with β-cell insulin content at ZT20

**Fig 3:**
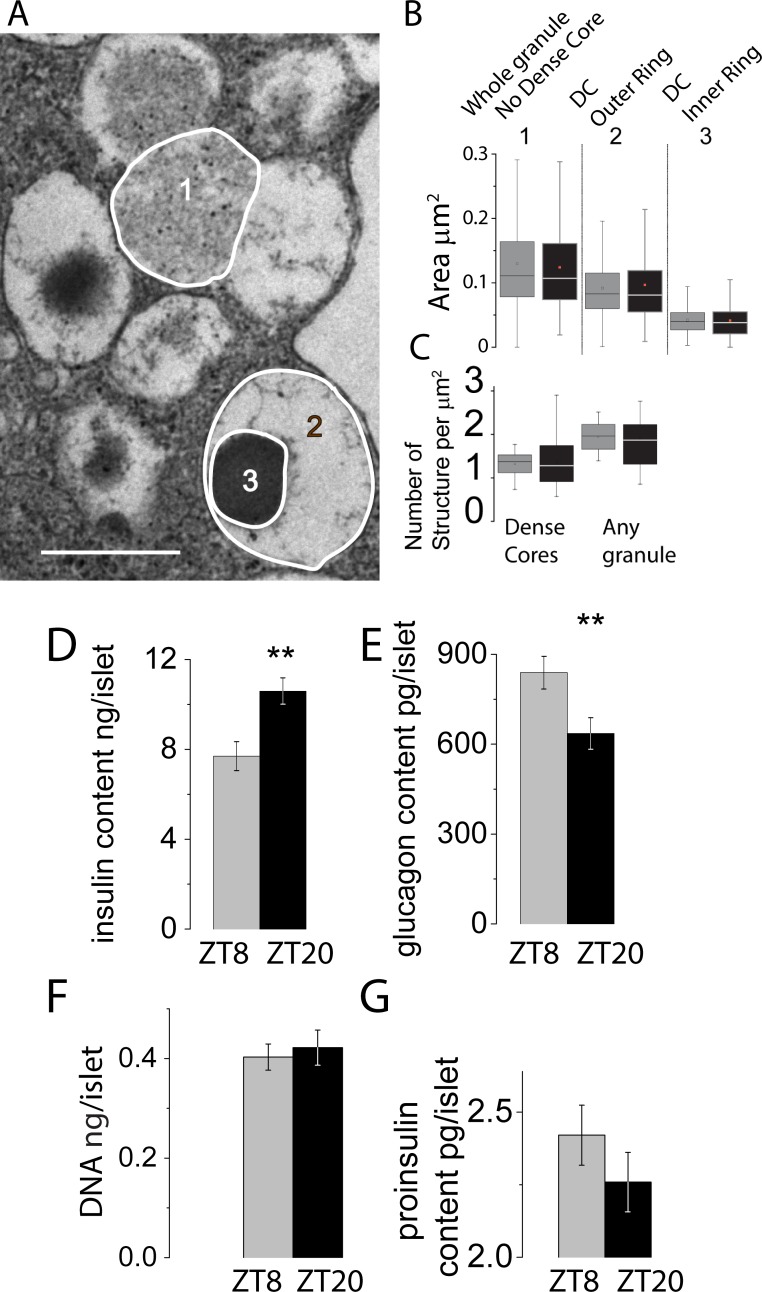
Subtle effects on β-cells ultrastructure and hormone content during the 24h cycle. *A*: Representative electron micrographs from β-cells and depiction of subgranule areas /granule types detected and analyzed. Scale bars: 0.5μm. *B* sizes and *C* numbers of region of interests (1, 2 and 3). Box plots groups of data according to their quartiles, of granule (1) and substructures areas (LDCV outer ring (2) or inner ring (3)) in ZT8 or ZT20 β-cells. The Mid line indicates the median whilst the small square within each box indicates the mean value. In panel C, data was averaged per cells. Panel D: mean insulin and (*E*:) glucagon content per islet ± S.E.M. for ZT8, n = 46 and 23 or ZT20, n = 45 and 22, respectively. Experimental replicates across a minimum of 6 mice per ZT. *P<0.05 using a *t-test*. *F*: mean DNA content per islet± S.E.M. for ZT8, n = 19 or ZT20, n = 23 replicates across a minimum of 3 mice per ZT. G: Proinsulin islet content in n = 57 for ZT8 islets (across 6 mice) and n = 57 for ZT20 islets (across 6 mice). Grey bars = ZT8; Dark bars = ZT20.

Ultrastructurally, islets from both ZT8 and ZT20 appeared identical (Fig 3A). The mean area of each type of granule (mature–presence- and immature -absence- of a dense core) were identical (0.127±0.07 μm^2^ and 0.1337±0.06 μm^2^ for immature granules and LDCV vesicles, respectively). Granule and dense core sizes were identical between ZT8 and ZT20 islets (Fig 3B). In agreement with capacitance data, the density of granules was similar between ZT8 and ZT20 islets (Fig 3C). Insulin content was increased 37% at ZT20 compared to ZT8 (Fig 3D). Interestingly, glucagon content was decreased by 34% (Fig 3E), and these changes were not correlated to islet DNA content (Fig 3F). Proinsulin content was marginally reduced (-7%, not significant) at ZT20 (Fig 3G).

### Islet gene expression profiles at ZT8 and ZT20

**Fig 4:**
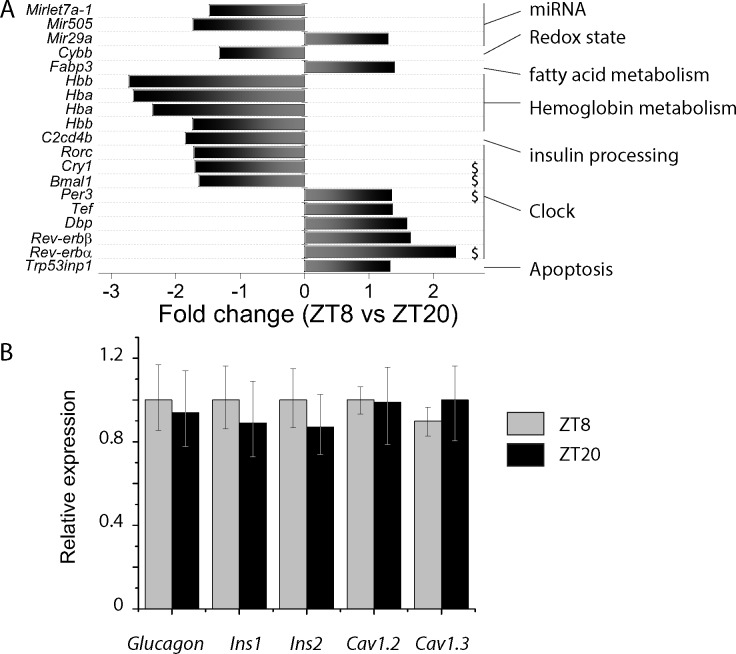
Expression of genes of interest at ZT8 and ZT20. *A*: Microarray data (GSE109882) unraveled a total of 301 transcripts significantly up or downregulated (see S1 Table for full details). 18 genes of interest (note that hemoglobin alpha and beta–Hba and Hbb- bore two probes on the microarray chip). Genes have been grouped by function. $ denotes genes identified in ref [14]. N = 5 experimental replicates for each group. B: relative expression of *glucagon*, *Ins1*, *Ins2*, *Cav1*.*2* and *Cav1*.*3* at ZT8 and ZT20 measured by qRT-PCR. Data are from 3 animals with 2 technical replicates per animal and per ZT.

A total of 301 genes were differentially expressed between ZT8 *vs* ZT20 islets (S1 Table, GSE109882). A total of 199 mRNA transcripts had higher levels at ZT8 (between 1.3- to 2.71-fold more than ZT20) and 102 had decreased levels (>1.3 fold). Given the focus of our study and the pertinent current literature, genes involved in exocytosis, insulin production and circadian rhythms were summarized in Fig 4A. Circadian genes *Rev-erbα*,*β* and *Bmal1* were differentially expressed as in Fig 1B. Several other genes were also detected. Specifically, *C2cd4b*, a gene involved in insulin maturation, was identified. In agreement with our capacitance data, no exocytotic genes were differentially expressed. In addition, *Ins1*, *Ins2* and *glucagon* and the subunits of the voltage-gated Calcium channels were not differentially expressed, what was subsequently confirmed by qRT-PCR (Fig 4B).

### Exploration of exocytosis at ZT12

**Fig 5:**
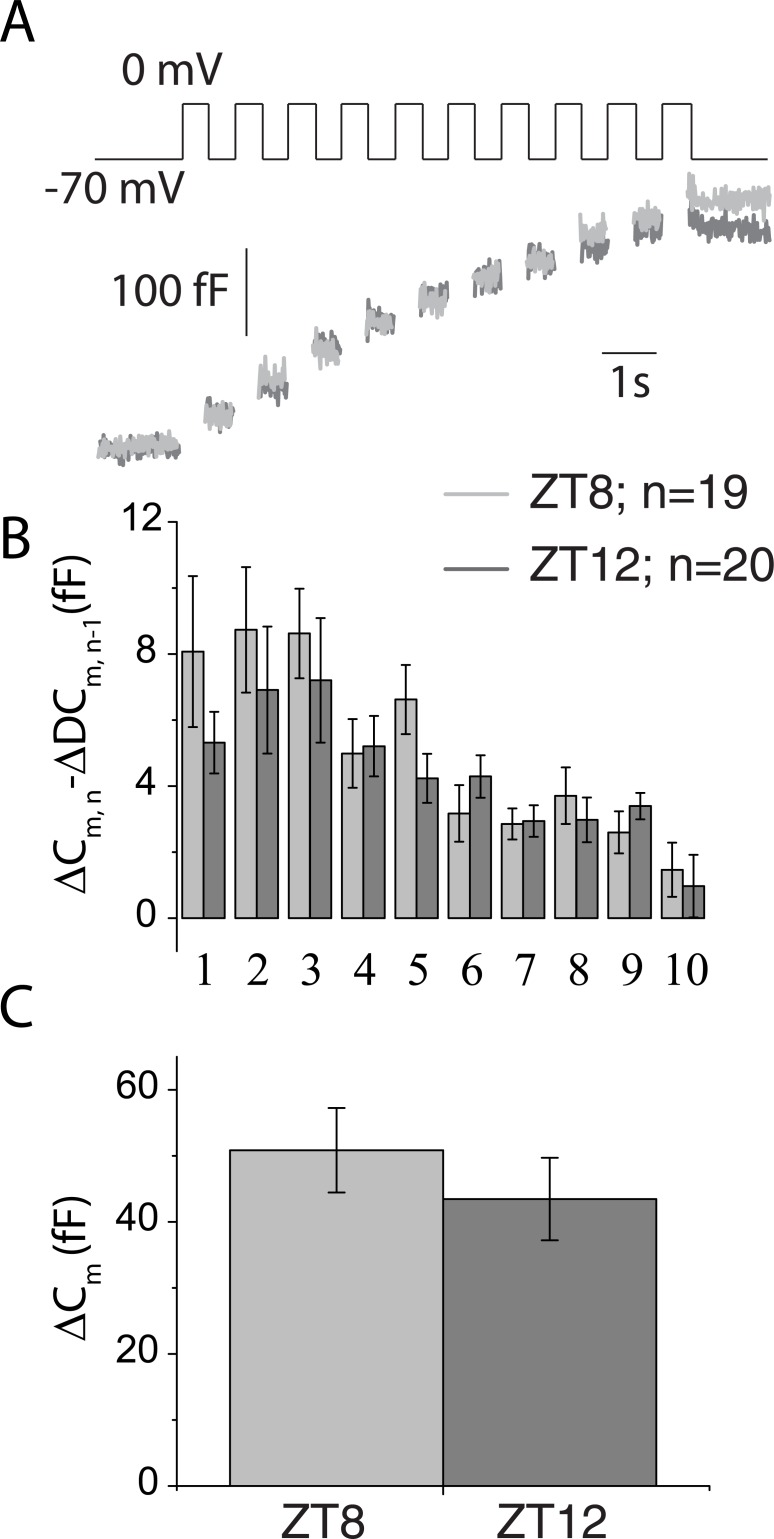
No change in exocytosis (measured via whole cell capacitance) between ZT8 and ZT12. *A*: representative traces of 10x500ms depolarization evoked capacitance increases. *B*: Step increases in membrane capacitance (Δ*C*_*m*_) for each depolarization pulse. C: Total increase in capacitance over the whole train. Whole cell patch clamps performed on single ZT8, n = 19 or ZT12, n = 20 β-cells across 2 mice per ZT. Bars are mean ± S.E.M. light grey = ZT8; Dark grey = ZT12 on all subpanels.

To verify if insulin exocytosis was upregulated at the beginning of the feeding period, capacitance measurements were repeated at ZT8 and ZT12. We found similar increases in depolarization-evoked capacitance measurements for ZT8 and ZT12 islets (Fig 5).

## Discussion

Several reports showed that the expression of genes involved in mitochondrial metabolism, exocytosis, granule processing and β-cell proliferation is circadian in both mouse [2, 4, 14, 15] and human [16] islets. Whilst changes in β-cell mass are outside the scope of the present study, our data argue that at certain points of the 24h cycle, β-cells adjust their daily secretory output solely via the modulation of intragranular insulin content. It is the first time that insulin exocytosis is measured directly by precise electrophysiological techniques at different time points of the 24h cycle. It is thus significant that despite a 21% increase in insulin secretion between ZT8 and ZT20, no changes in the number of fusion events were detected. Likewise, calcium fluorimetry and precise analysis of voltage-gated calcium channels via electrophysiology and qRT-PCR showed that glucose metabolism and ATP induced closure of Ca^2+^ channels could not account for these differences. Instead, as evidenced by ultrastructural data and islet insulin content, the modulation of insulin production and packaging underlined these effects. Insulin content was measured using an elisa kit that does not cross react with C-peptide or pro-insulin. Immunoreactive insulin is matured in granules so what we are measuring is whole granule insulin content. This is why the lack of differences in granule number measured by EM is so important. The size of the granules measured by EM is also important in relation to capacitance measurements which are actually measuring the size of vesicle fusing with the plasma membrane. The lack of differences in capacitance measurements at ZT8 and ZT20 in full view of the ultrastructural data (same size) unambiguously argues that the same number of vesicles are fusing at ZT8 and ZT20. If the granule density and size are the same at ZT20 and ZT8, whilst insulin is augmented at ZT20, it can only mean that insulin granular content is increased at this time point.

It is important to remember these findings were established in islets cultured for 24h. At this stage, it can be anticipated that the metabolic status of islets is identical between the two groups, which ensures that the resulting phenotype is only due to the 24h cycle rather than metabolic cues. The beta cell is thus capable, over time, of changing the amount of insulin liberated by each granule, making the number of exocytotic events perhaps less relevant in controlling insulin release at this time point. This is further supported by the fact that both basal and glucose induced insulin secretion were increased between ZT8 and ZT20. Compelling evidence that modulation of insulin content is physiologically relevant is the fact that glucagon content followed the inversed pattern.

The insulin gene has equally been reported to be circadian in expression [5] or remain stable [2]. Using a siRNA against the clock gene Rev-erb α, insulin expression was marginally upregulated whilst insulin content was concomitantly reduced [15]. Interestingly, in human islets from normal and diabetic donors, PER2, PER3 and CRY2 expression levels correlated positively with insulin content [17]. On the other hand, a β-cell specific Bmal1 KO bore no effect on insulin content [18]. These discrepancies may come from 1) lack of power in detecting the effect since measuring islet insulin content reliably can be challenging making it prone to “false negatives”; and 2) the fact that it has different kinetics than gene expression. The latter is illustrated by previous studies [5] showing that *Ins1* and *Ins2* transcriptional activities peak briefly at ZT12, whilst increase in blood insulin levels are only detected 4h later. Accordingly, *Ins1* and *Ins2* mRNA transcripts have low expression at the two time points used in our study (ZT8 and ZT20); and proinsulin levels were only marginally reduced at ZT20, suggesting that insulin processing had most likely peaked just beforehand. *C2cd4b*, a gene implicated in diabetes and proinsulin to insulin conversion via a genome-wide association study [19], was differentially expressed but the cellular function and relevance of *C2cd4b* to diabetes has yet to be validated [20].

The aim of our paper was to investigate the physiological mechanisms underlying the modulation of insulin secretion between day and night. Our transcriptomic data is only a snapshot of two time points and should be regarded as a minor addition to the wealth of data in transcriptomic studies done over the full cycle. Nevertheless, in addition to *C2cd4b*, we identified two additional genes regulated over time, such as the hemoglobin (*Hb*) genes. *Hb* expression has been reported in nonerythroid cells, but it is the first time that it is reported in islet cells. Coincidentally, ZT8 and ZT20 correspond to the maximal and minimal relative expression of Rev-erb α, respectively. REV-ERBs function as physiological sensors of intracellular heme and the intracellular heme undergoes circadian regulation [21] allowing REV-ERB proteins to modulate repression of their target genes and to shape the amplitude of the circadian rhythm.

Interestingly, the ~4h delay between insulin genes expression and availability [5] suggests that mice would require another mechanism for the secretion of appropriate amounts of insulin at ZT12 (i.e. the beginning of the feeding period). Could this be exocytosis? Whole genome transcriptomics over the circadian cycle have indeed detected exocytotic or granule trafficking genes as being differentially expressed [2, 14, 15, 22]; however, it has long been established that insulin sensitivity is drastically increased at ZT12 in rats and that insulin secretion rates *in vivo* only augment throughout the day in humans [23]. Our additional measurements show that exocytosis is not upregulated at ZT12. If anything, a trend for reduction in exocytosis was apparent in the first steps of depolarization at ZT12. It should be noted that capacitance measurements are done in a cell configuration that clamps cAMP and ATP levels. Effects on exocytosis mediated by these biological active molecules are thus not measured, and the recent publication of *Ucp2* as a circadian gene together with measures of ATP production showing a circadian pattern may be pertinent at ZT12 [4]. *Ucp2* involvement was mainly studied at ZT4 and ZT14. At the main time points of our experimental paradigm, ATP is however unlikely to play a role: After verifying that Voltage gated Ca^2+^ channels electrophysiological properties were unaffected at ZT8 and ZT20, we measured glucose induced [Ca^2+^]i transients which are indirectly linked to ATP production. The lack of differences in the amplitude or period of fast Ca^2+^ oscillations argues that mitochondrial function is identical at ZT8 and ZT20. We have previously published that mitochondrial reactive oxygen species are obligatory signals for glucose induced insulin secretion. These signals do not require ATP for modulating insulin secretion and impact on [Ca^2+^]_i_ [24]. In this context, modulation of *Ucp2* gene expression could have unsuspected effects on ROS production independent of ATP and impact on insulin secretion. But it is important to remember that basal insulin secretion (not just GSIS) was also increased at ZT20 and this is more likely due to changes in granule insulin content especially since [Ca^2+^]_i_ levels, granule density and exocytosis were unaffected. Furthermore, the 21% late night increases in insulin secretion (ZT20) would be fully accounted for by the 37% increase in insulin content and would not require another mechanism.

Our results also call for a better understanding of how insulin content regains basal values during the resting phase. The ZT groups used in our study are independent and as such, reflect either the increase in insulin content between ZT8 and ZT20 or the decrease between ZT20 and ZT8. It has been demonstrated that the half-life of the insulin protein exceeds 20h [25] however, newly synthetized insulin is preferentially secreted whilst old granules show reduced microtubule-dependent mobility [26] and are eventually stored and degraded. The drop in insulin content between ZT20 and ZT8 (12h) reaches 37% in our experimental paradigm and although the time frame is short, we hypothesize that the preferential liberation of newly synthetized ‘insulin rich” granules between ZT20 and ZT8 fully accounts for a return to basal values. Furthermore, a novel mechanism which specifically degrades nascent insulin granules during extreme fasting has been unraveled [27]. It has not been investigated whether this pathway changes over the 24h cycle; we did not see evidence of this via electron microscopy, however.

## Conclusions

Our report shows that upregulation of insulin secretion to face metabolic demands during the feeding period at ZT20 is solely accounted for by increased insulin granule content. In the context of other studies which have reported modulation of exocytosis, metabolism and granule trafficking at other time points, our results show that the regulation of the beta cell over the 24h cycle is remarkably complex and that specific pathways all involved in modulating the beta cell output are finely regulated according to the time of the day. This has implications in islet research as it may explain discrepancies between studies where experimental procedures are carried out at different time points.

## Supporting information

S1 Fig Increasing culture length negatively impacts on amplitude of voltage gated channel activities. (A) Islets were collected from normal cycle mice at 09:00 on Day 0 (ZT2) and dissociated cells put in culture. Voltage gated channel activities were measured at 15:00 of Day 1 (culture length 30h ZT8) and 03:00 on Day 2. (B) On an inverted set of mice, the procedure was repeated. *: p>0.05 using ANOVA repeated measures.
(DOCX)

S1 Table Probes used in qRTPCR. (DOCX)

S1 Table Effects of circadian rhythm on gene expression. (DOCX)

## Abbreviations

LDCV: Large Dense Core Vesicles
GSIS: Glucose Stimulated Insulin Secretion
