# Supplementary material for: Modulation of large dense core vesicle insulin content mediates rhythmic hormone release from pancreatic beta cells over the 24h cycle
Source: PLoS One. 2018 Mar 15;13(3):e0193882. doi: 10.1371/journal.pone.0193882 (PMC5854349; doi:10.1371/journal.pone.0193882)
Supplement: S1 Fig — (A) Islets were collected from normal cycle mice at 09:00 on Day 0 (ZT2) and dissociated cells put in culture. Voltage gated channel activities were measured at 15:00 of Day 1 (culture length 30h ZT8) and 03:00 on Day 2. (B) On an inverted set of mice, the procedure was repeated. *: p>0.05 using ANOVA repeated measures. (DOCX) [file pone.0193882.s001.docx]

**S1 Fig. Increasing culture length negatively impacts on amplitude of Voltage gated channel activities.** (A) Islets were collected from normal cycle mice at 09:00 on Day 0 (ZT2) and dissociated cells put in culture. Voltage gated channel activities were measured at 15:00 of Day 1 (culture length 30h ZT8) and 03:00 on Day 2. (B) On an inverted set of mice, the procedure was repeated. *: p>0.05 using ANOVA repeated measures.

**
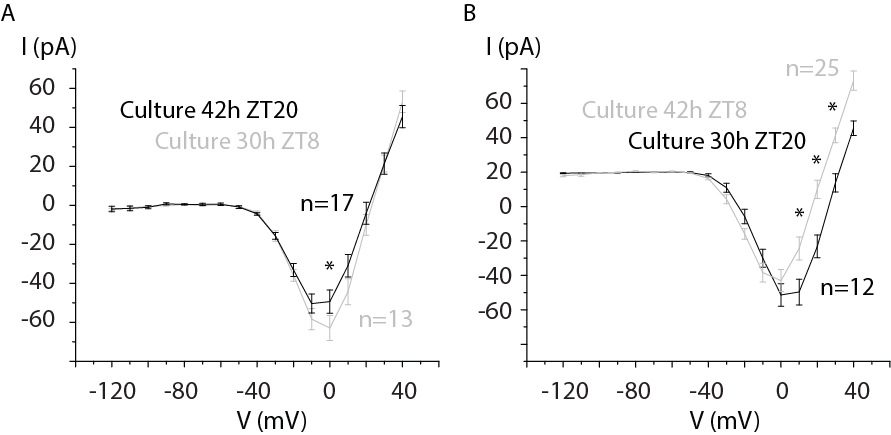
**
