# Supplementary material for: Modulation of large dense core vesicle insulin content mediates rhythmic hormone release from pancreatic beta cells over the 24h cycle
Source: PLoS One. 2018 Mar 15;13(3):e0193882. doi: 10.1371/journal.pone.0193882 (PMC5854349; doi:10.1371/journal.pone.0193882)
Supplement: S1 Table — (DOCX) [file pone.0193882.s002.docx]

**S1 Table.** Probes used in qRTPCR

|  | gene symbol | Taqman assay |  |
| --- | --- | --- | --- |
| *Rev-erb α* | Nr1d1 | Mm00520708_m1 |  |
| *Rev-erb β* | Nr1d2 | Mm00441730_m1 |  |
| *Bmal1* | Arntl | Mm00500226_m1 |  |
| *36B4* | Rplp0 | Mm00725448_s1 |  |
| *Ins1* | Ins1 | Mm01950294_s1 |  |
| *Ins2* | Ins2 | Mm00731595_gH |  |
| *Gcg* | Gcg | Mm01269055_m1 |  |
