# Supplementary material for: Modulation of large dense core vesicle insulin content mediates rhythmic hormone release from pancreatic beta cells over the 24h cycle
Source: PLoS One. 2018 Mar 15;13(3):e0193882. doi: 10.1371/journal.pone.0193882 (PMC5854349; doi:10.1371/journal.pone.0193882)
Supplement: S1 Table — (DOCX) [file pone.0193882.s003.docx]

**S2 Table.** Effects of circadian rhythm on gene expression.

GCRMA (Guanine Cytosine Robust Multi-Array Analysis) normalized differentially expressed genes with *P*≤0.05 (Welch t-test) and a ≥1.3-fold differential expression between ZT8 and ZT20 islets. Lists the genes with increased or reduced expression in ZT20. The microarray was carried out using RNA prepared from islets of 10-weeks old mice (5 animals per genotype).

| **Fold Change (linear)** | **ANOVA p-value (Condition1 vs. Condition2)** | **Description** | **Gene Symbol** |
| --- | --- | --- | --- |
| *increase* |  |  |  |
| **2.71** | 0.038708 | microRNA 5123 | Mir5123 |
| **2.35** | 0.000007 | nuclear receptor subfamily 1, group D, member 1 | Nr1d1 |
| **1.99** | 0.00987 | predicted gene, 26870 | Gm26870 |
| **1.84** | 0.027883 | small nucleolar RNA, C/D box 70 | Snord70 |
| **1.8** | 0.011846 | mitochondrially encoded tRNA glycine | mt-Tg |
| **1.79** | 0.026376 | X-linked lymphocyte-regulated 3D, pseudogene | Xlr3d-ps |
| **1.78** | 0.037791 | small nucleolar RNA, C/D box 98 | Snord98 |
| **1.73** | 0.038988 | small nucleolar RNA, C/D box 42A | Snord42a |
| **1.72** | 0.047065 | X-linked lymphocyte-regulated 3A | Xlr3a |
| **1.71** | 0.045481 | X-linked lymphocyte-regulated 3B | Xlr3b |
| **1.7** | 0.029062 | X-linked lymphocyte-regulated 3C | Xlr3c |
| **1.68** | 0.002641 | predicted gene, 21833 | Gm21833 |
| **1.67** | 0.040988 | X-linked lymphocyte-regulated 3E, pseudogene | Xlr3e-ps |
| **1.65** | 0.002697 | nuclear receptor subfamily 1, group D, member 2 | Nr1d2 |
| **1.62** | 0.001312 | predicted gene 4883 | Gm4883 |
| **1.61** | 0.002603 | predicted gene 14989; SEC61, gamma subunit (Sec61g) pseudogene | Gm14989 |
| **1.59** | 0.000486 | D site albumin promoter binding protein | Dbp |
| **1.58** | 0.002732 | predicted gene 16550; DnaJ homolog subfamily C (Dnajc9) pseudogene | Gm16550 |
| **1.56** | 0.000023 | predicted gene 4899 | Gm4899 |
| **1.56** | 0.005913 | predicted gene, 23767 | Gm23767 |
| **1.56** | 0.000094 | ribosomal protein L9, pseudogene 1 | Rpl9-ps1 |
| **1.55** | 0.013657 | predicted gene, 24276 | Gm24276 |
| **1.54** | 0.016188 | brain cytoplasmic RNA 1 | Bc1 |
| **1.54** | 0.023834 | mitochondrially encoded tRNA arginine | mt-Tr |
| **1.54** | 0.013374 | predicted gene, 25909 | Gm25909 |
| **1.53** | 0.001671 | microRNA 684-1 | Mir684-1 |
| **1.53** | 0.001671 | microRNA 684-1 | Mir684-1 |
| **1.53** | 0.001671 | microRNA 684-1 | Mir684-1 |
| **1.53** | 0.001671 | microRNA 684-1 | Mir684-1 |
| **1.53** | 0.001671 | microRNA 684-1 | Mir684-1 |
| **1.53** | 0.001243 | predicted gene 12142; basic transcription factor 3 (Btf3) pseudogene | Gm12142 |
| **1.53** | 0.003807 | predicted gene, 24588 | Gm24588 |
| **1.51** | 0.001891 | microRNA 684-2 | Mir684-2 |
| **1.51** | 0.039154 | predicted gene 13785; 40S ribosomal protein S6 (Rps6) pseudogene | Gm13785 |
| **1.51** | 0.000675 | predicted gene, 22015 | Gm22015 |
| **1.5** | 0.006901 | predicted gene, 22694 | Gm22694 |
| **1.5** | 0.052453 | predicted gene, 23862 | Gm23862 |
| **1.49** | 0.001878 | microRNA 684-1 | Mir684-1 |
| **1.49** | 0.034977 | predicted gene 11114 | Gm11114 |
| **1.48** | 0.000075 | predicted gene 10770 | Gm10770 |
| **1.48** | 0.000368 | predicted gene 15289; electron transferring flavoprotein, alpha polypeptide (Etfa) pseudogene | Gm15289 |
| **1.47** | 0.01491 | predicted gene 10780 | Gm10780 |
| **1.47** | 0.036952 | predicted gene, 24856 | Gm24856 |
| **1.46** | 0.000866 | predicted gene, 17377 | Gm17377 |
| **1.46** | 0.037179 | predicted gene, 25559 | Gm25559 |
| **1.46** | 0.001586 | predicted pseudogene 10224; 60S ribosomal protein L15 (Rpl15) pseudogene | Gm10224 |
| **1.46** | 0.019223 | S-adenosylmethionine decarboxylase 2 | Amd2 |
| **1.45** | 0.002106 | predicted gene, 22030 | Gm22030 |
| **1.44** | 0.004204 | predicted gene, 17511 | Gm17511 |
| **1.44** | 0.047354 | predicted gene, 22188 | Gm22188 |
| **1.44** | 0.018272 | predicted pseudogene 6654; ribosomal protein S26 (Rps26) pseudogene | Gm6654 |
| **1.43** | 0.01866 | microRNA 28c | Mir28c |
| **1.43** | 0.001002 | predicted gene 14284; small nuclear ribonucleoprotein E (Snrpe) pseudogene | Gm14284 |
| **1.43** | 0.00695 | predicted gene 15013 | Gm15013 |
| **1.43** | 0.048058 | predicted gene, 25101 | Gm25101 |
| **1.42** | 0.00774 | fatty acid binding protein 3, muscle and heart, pseudogene 1 | Fabp3-ps1 |
| **1.42** | 0.001729 | predicted gene 11849; pseudogene similar to part of ribosomal protein L21 (Rpl21) | Gm11849 |
| **1.42** | 0.001798 | predicted gene 14409; novel KRAB box and zinc finger, C2H2 type domain containing protein | Gm14409 |
| **1.42** | 0.000729 | predicted gene 5093 | Gm5093 |
| **1.42** | 0.006398 | predicted gene, 21836 | Gm21836 |
| **1.42** | 0.000699 | predicted pseudogene 16373; enhancer of yellow 2 homolog (Drosophila) (Eny2) pseudogene | Gm16373 |
| **1.41** | 0.053036 | microRNA 467e | Mir467e |
| **1.41** | 0.003742 | predicted gene 13167; high mobility group box 2 (Hmgb2) pseudogene (Gm13167) | Gm13167 |
| **1.41** | 0.000665 | predicted gene 13215; ribosomal protein S27a (Rps27a) pseudogene | Gm13215 |
| **1.41** | 0.003101 | predicted gene 14534; peptidylprolyl isomerase A (Ppia) pseudogene | Gm14534 |
| **1.41** | 0.009485 | predicted gene 17132; L1 element, A subfamily pseudogene | Gm17132 |
| **1.41** | 0.001055 | predicted gene 6335; DnaJ (Hsp40) homolog, subfamily A member 1 (Dnaja1) pseudogene | Gm6335 |
| **1.41** | 0.043638 | predicted gene, 25672 | Gm25672 |
| **1.4** | 0.010721 | fatty acid binding protein 3, muscle and heart | Fabp3 |
| **1.4** | 0.00028 | predicted gene 12482; nascent polypeptide-associated complex alpha polypeptide (Naca) pseudogene | Gm12482 |
| **1.39** | 0.0009 | 60S ribosomal protein L17, pseudogene; predicted pseudogene 10166; ribosomal protein L17 (Rpl17) pseudogene | Gm10166 |
| **1.39** | 0.015516 | predicted gene 10933 | Gm10933 |
| **1.39** | 0.00007 | predicted gene 12261; high mobility group box 1 pseudogene | Gm12261 |
| **1.39** | 0.000514 | predicted gene 13231; SWI/SNF related, matrix associated, actin dependent regulator of chromatin, subfamily a, member 5 (Smarca5) pseudogene | Gm13231 |
| **1.39** | 0.008892 | predicted gene 14894; pseudogene similar to part of ring finger and WD repeat domain 2 Rfwd2 | Gm14894 |
| **1.39** | 0.005405 | predicted gene, 22508 | Gm22508 |
| **1.39** | 0.025508 | predicted gene, 23137 | Gm23137 |
| **1.38** | 0.002985 | microRNA 703 | Mir703 |
| **1.38** | 0.027559 | mitochondrially encoded tRNA methionine; NADH dehydrogenase subunit 2; mitochondrially encoded tRNA tryptophan | mt-Tm; ND2; mt-Tw |
| **1.38** | 0.000187 | predicted gene 10139 | Gm10139 |
| **1.38** | 0.001127 | predicted gene 13396; ring finger and WD repeat domain 2 (Rfwd2) pseudogene | Gm13396 |
| **1.38** | 0.000282 | predicted gene 14405; novel zinc finger protein pseudogene | Gm14405 |
| **1.38** | 0.010448 | predicted gene, 24456 | Gm24456 |
| **1.38** | 0.030915 | predicted gene, 25312 | Gm25312 |
| **1.38** | 0.007213 | predicted gene, 27038; ribosomal protein S2 (Rps2) pseudogene | Gm27038 |
| **1.38** | 0.039751 | zinc finger protein 51 pseudogene; predicted gene 10226; predicted gene 10509 | Gm10509; Gm10226 |
| **1.37** | 0.04192 | high-mobility group nucleosome binding domain 5 | Hmgn5 |
| **1.37** | 0.001618 | predicted gene 10972 | Gm10972 |
| **1.37** | 0.007649 | predicted gene 11204; pseudogene similar to part of chondroitin sulfate proteoglycan 6 Cspg6 | Gm11204 |
| **1.37** | 0.004657 | predicted gene 14335; TAF9 RNA polymerase II TATA box binding protein TBP-associated factor (Taf9) pseudogene | Gm14335 |
| **1.37** | 0.001138 | predicted gene 9431; heterogeneous nuclear ribonucleoprotein A3 (Hnrpa3) pseudogene | Gm9431 |
| **1.37** | 0.009054 | predicted gene, 22151 | Gm22151 |
| **1.37** | 0.005918 | predicted gene, 25040 | Gm25040 |
| **1.37** | 0.000869 | ribosomal protein L10 (Rpl10) pseudogene | RP23-13G1.6 |
| **1.37** | 0.002533 | thyrotroph embryonic factor | Tef |
| **1.36** | 0.015911 | period circadian clock 3; period homolog 3 (Drosophila); period homolog 3 (Drosophila) (Per3), mRNA. | Per3 |
| **1.36** | 0.001506 | predicted gene 10268 | Gm10268 |
| **1.36** | 0.000301 | predicted gene 11008; novel transcript | Gm11008 |
| **1.36** | 0.000991 | predicted gene 12129; heterogeneous nuclear ribonucleoprotein pseudogene | Gm12129 |
| **1.36** | 0.001959 | predicted gene 13136; SWI/SNF related, matrix associated, actin dependent regulator of chromatin, subfamily a, member 1 (Smarca1) pseudogene (Gm13136) | Gm13136 |
| **1.36** | 0.000263 | predicted gene 14421; novel zinc finger protein pseudogene | Gm14421 |
| **1.36** | 0.000386 | predicted gene 16436; karyopherin (importin) alpha 2 (Kpna2) pseudogene | Gm16436 |
| **1.36** | 0.000674 | predicted gene 6767; eukaryotic translation elongation factor 1 alpha 1 (Eef1a1) pseudogene | Gm6767 |
| **1.36** | 0.000342 | predicted gene 7381 | Gm7381 |
| **1.36** | 0.000056 | predicted gene, 17404 | Gm17404 |
| **1.36** | 0.017841 | predicted pseudogene 5356 | Gm5356 |
| **1.36** | 0.000824 | ribosomal protein S15A, pseudogene 5 | Rps15a-ps5 |
| **1.36** | 0.035553 | RNA, Y1 small cytoplasmic, Ro-associated | Rny1 |
| **1.35** | 0.022736 | microRNA 466f-3 | Mir466f-3 |
| **1.35** | 0.011341 | predicted gene 11512; cytochrome c oxidase subunit I (COX1) pseudogene | Gm11512 |
| **1.35** | 0.03369 | predicted gene 13080; trafficking protein particle complex 2 (Trappc2) pseudogene | Gm13080 |
| **1.35** | 0.003458 | predicted gene 13831; enhancer of rudimentary homolog (Drosophila) (Erh) pseudogene | Gm13831 |
| **1.35** | 0.006973 | predicted gene 14407; ribosomal protein L27a (Rpl27a) pseudogene | Gm14407 |
| **1.35** | 0.008997 | predicted gene 15065; ring finger and WD repeat domain 2 (Rfwd2) pseudogene | Gm15065 |
| **1.35** | 0.027447 | predicted gene 15610; coiled-coil domain containing 72 (Ccdc72) pseudogene | Gm15610 |
| **1.35** | 0.000095 | predicted gene 16139; ribosomal protein S27a (Rps27a) pseudogene | Gm16139 |
| **1.35** | 0.001607 | predicted gene 16200; ribosomal protein L35a (Rpl35a) pseudogene | Gm16200 |
| **1.35** | 0.016659 | predicted gene, 17555 | Gm17555 |
| **1.35** | 0.027528 | predicted gene, 23732 | Gm23732 |
| **1.35** | 0.000486 | predicted gene, 23876 | Gm23876 |
| **1.35** | 0.001436 | predicted gene, 23881 | Gm23881 |
| **1.35** | 0.027528 | predicted gene, 25173 | Gm25173 |
| **1.35** | 0.027528 | predicted gene, 25211 | Gm25211 |
| **1.35** | 0.027528 | predicted gene, 26324 | Gm26324 |
| **1.34** | 0.018025 | eukaryotic translation initiation factor 3, subunit 6, pseudogene 2 | Eif3s6-ps2 |
| **1.34** | 0.000909 | predicted gene 13135; SWI/SNF related, matrix associated, actin dependent regulator of chromatin, subfamily a pseudogene (Gm13135) | Gm13135 |
| **1.34** | 0.046769 | predicted gene 13210; ring finger and WD repeat domain 2 (Rfwd2) pseudogene | Gm13210 |
| **1.34** | 0.005169 | predicted gene 13339; NADH dehydrogenase subunit 2 (ND2) pseudogene | Gm13339 |
| **1.34** | 0.001212 | predicted gene 14681; nucleophosmin 1 (Npm1) pseudogene | Gm14681 |
| **1.34** | 0.000144 | predicted gene 3466; ribosomal protein L3 (Rpl3) pseudogene | Gm3466 |
| **1.34** | 0.001749 | predicted gene 6712 | Gm6712 |
| **1.34** | 0.006423 | predicted gene, 21897 | Gm21897 |
| **1.34** | 0.005543 | predicted gene, 22776 | Gm22776 |
| **1.34** | 0.052015 | predicted gene, 23375 | Gm23375 |
| **1.33** | 0.013952 | acidic (leucine-rich) nuclear phosphoprotein 32 family, pseudogene | Anp32-ps |
| **1.33** | 0.007478 | histone cluster 1, H2ak; histone 1, H2ak | Hist1h2ak |
| **1.33** | 0.012984 | predicted gene 12852; ribosomal protein S4 (Rps4) X-linked pseudogene | Gm12852 |
| **1.33** | 0.000657 | predicted gene 14541; ribosomal protein L39 (Rpl39) pseudogene | Gm14541 |
| **1.33** | 0.001216 | predicted gene 14670; ring finger and WD repeat domain 2 (Rfwd2) pseudogene | Gm14670 |
| **1.33** | 0.005308 | predicted gene 14897; pseudogene similar to part of YME1-like 1 (S. cerevisiae) Yme1l1 | Gm14897 |
| **1.33** | 0.00511 | predicted gene 15497; ribosomal protein L17 (Rpl17) pseudogene | Gm15497 |
| **1.33** | 0.001295 | predicted gene 15878; Novel pseudogene | Gm15878 |
| **1.33** | 0.000888 | predicted gene 6712 | Gm6712 |
| **1.33** | 0.028842 | predicted gene 7598; putative novel transcript | Gm7598 |
| **1.33** | 0.001083 | predicted gene 9308; basic transcription factor 3 (Btf3) pseudogene | Gm9308 |
| **1.33** | 0.016014 | predicted gene, 25271 | Gm25271 |
| **1.33** | 0.002034 | predicted pseudogene 5457 | Gm5457 |
| **1.33** | 0.030258 | transformation related protein 53 inducible nuclear protein 1 | Trp53inp1 |
| **1.32** | 0.004355 | developmentally regulated GTP binding protein 1 (Drg1) pseudogene | RP23-265F16.5 |
| **1.32** | 0.026059 | peptidylprolyl isomerase (cyclophilin)-like 6 | Ppil6 |
| **1.32** | 0.001149 | predicted gene 11759; ribosomal protein S27a (Rps27a) pseudogene | Gm11759 |
| **1.32** | 0.001622 | predicted gene 12372; TAF9 RNA polymerase II TATA box binding protein (TBP)-associated factor (Taf9) pseudogene | Gm12372 |
| **1.32** | 0.031878 | predicted gene 13419; ring finger and WD repeat domain 2 (Rfwd2) pseudogene | Gm13419 |
| **1.32** | 0.001571 | predicted gene 13430; SMT3 suppressor of mif two 3 homolog 2 (yeast) (Sumo2) pseudogene; microRNA 684-1 | Gm13430; Mir684-1 |
| **1.32** | 0.005694 | predicted gene 14650; ribosomal protein L6 (Rpl6) pseudogene | Gm14650 |
| **1.32** | 0.002456 | predicted gene 16125; eukaryotic translation initiation factor 1 (Eif1) pseudogene | Gm16125 |
| **1.32** | 0.000285 | predicted gene 7027; ribosomal protein L6 (Rpl6) pseudogene | Gm7027 |
| **1.32** | 0.000106 | predicted gene, 17449 | Gm17449 |
| **1.32** | 0.019876 | predicted gene, 23610 | Gm23610 |
| **1.32** | 0.035946 | predicted gene, 24729 | Gm24729 |
| **1.32** | 0.050621 | predicted gene, 27033; novel transcript | Gm27033 |
| **1.32** | 0.003134 | ribisomal protein L5, pseudogene 2; predicted gene 14217; ribosomal protein L5 (Rpl5) pseudogene | Rpl5-ps2; Gm14217 |
| **1.32** | 0.002391 | ribosomal protein S6, pseudogene 4; ribosomal protein S6 (Rps6) pseudogene | Rps6-ps4 |
| **1.31** | 0.001409 | centromere protein Q | Cenpq |
| **1.31** | 0.001415 | predicted gene 11473; ribosomal protein S16 (Rps16) pseudogene | Gm11473 |
| **1.31** | 0.007268 | predicted gene 11581; mortality factor 4 (MORF4) pseudogene | Gm11581 |
| **1.31** | 0.005191 | predicted gene 11604; ribosomal protein S6 (Rps6) pseudogene | Gm11604 |
| **1.31** | 0.003907 | predicted gene 14392; novel zinc finger protein pseudogene | Gm14392 |
| **1.31** | 0.000604 | predicted gene 14400; novel zinc finger protein pseudogene | Gm14400 |
| **1.31** | 0.001909 | predicted gene 15151; upregulated during skeletal muscle growth 5 (Usmg5) pseudogene | Gm15151 |
| **1.31** | 0.003493 | predicted gene 17131; viral polymerase like LINE element pseudogene | Gm17131 |
| **1.31** | 0.030682 | predicted gene 6645; transmembrane and tetratricopeptide repeat containing 3 (Tmtc3) pseudogene | Gm6645 |
| **1.31** | 0.000168 | predicted gene 8217; ribosomal protein L23a (Rpl23a) pseudogene | Gm8217 |
| **1.31** | 0.019572 | predicted gene, 22421 | Gm22421 |
| **1.31** | 0.017248 | predicted gene, 26980; H3 histone, family 3A (H3f3a) pseudogene | Gm26980 |
| **1.31** | 0.000092 | predicted pseudogene 10163; ribosomal protein L21 (Rpl21) pseudogene | Gm10163 |
| **1.31** | 0.000914 | predicted pseudogene 6142; tubulin cofactor a (Tbca) pseudogene | Gm6142 |
| **1.3** | 0.021691 | microRNA 29a | Mir29a |
| **1.3** | 0.008919 | predicted gene 11794; constitutive photomorphogenic protein 1 (Cop1) pseudogene | Gm11794 |
| **1.3** | 0.010457 | predicted gene 12947; ribosomal protein S15 (Rps15) pseudogene | Gm12947 |
| **1.3** | 0.000644 | predicted gene 13140; SWI/SNF related, matrix associated, actin dependent regulator of chromatin, subfamily a, member 5 (Smarca5) pseudogene (Gm13140) | Gm13140 |
| **1.3** | 0.010483 | predicted gene 14412; novel KRAB box and zinc finger, C2H2 type domain containing protein | Gm14412 |
| **1.3** | 0.000344 | predicted gene 14416; novel zinc finger protein | Gm14416 |
| **1.3** | 0.002984 | predicted gene 15733; Ribosomal protein L15 (Rpl15) pseudogene | Gm15733 |
| **1.3** | 0.006443 | predicted gene 15808; Y box protein 1 (Ybx1) pseudogene | Gm15808 |
| **1.3** | 0.003017 | predicted gene 16111; ribosomal protein L17 (Rpl17) pseudogene | Gm16111 |
| **1.3** | 0.002382 | predicted gene 16363; novel zinc finger protein pseudogene | Gm16363 |
| **1.3** | 0.000923 | predicted gene 17014; ribosomal protein L9 (Rpl9) pseudogene | Gm17014 |
| **1.3** | 0.017327 | predicted gene 8532 | Gm8532 |
| **1.3** | 0.00372 | predicted gene, 17631 | Gm17631 |
| **1.3** | 0.00744 | predicted gene, 21750 | Gm21750 |
| **1.3** | 0.013328 | predicted gene, 23144 | Gm23144 |
| **1.3** | 0.007577 | predicted gene, 24149 | Gm24149 |
| **1.3** | 0.010746 | predicted gene, 25937 | Gm25937 |
| **1.3** | 0.038546 | predicted gene, 26250 | Gm26250 |
| **1.3** | 0.00084 | predicted pseudogene 5396; high mobility group box 1(Hmgb1) pseudogene | Gm5396 |
| **1.3** | 0.001796 | predicted pseudogene 7123; ribosomal protein L38 (Rpl38) pseudogene | Gm7123 |
| **1.3** | 0.002354 | ribosomal protein S15, pseudogene 2 | Rps15-ps2 |
| **1.3** | 0.050577 | RNA, Y1 small cytoplasmic, Ro-associated | Rny1 |
| *Decrease* |  |  |  |
| **-1.3** | 0.004841 | Ighv5-12-4 immunoglobulin heavy variable 5-12-4 | Ighv5-12-4 |
| **-1.3** | 0.027212 | olfactory receptor 1015 | Olfr1015 |
| **-1.3** | 0.004512 | olfactory receptor 122 | Olfr122 |
| **-1.3** | 0.009064 | predicted gene 12801; putative novel transcript | Gm12801 |
| **-1.3** | 0.001044 | predicted gene, 23308 | Gm23308 |
| **-1.3** | 0.003836 | predicted gene, 24505 | Gm24505 |
| **-1.3** | 0.003489 | predicted gene, 24741 | Gm24741 |
| **-1.3** | 0.050378 | predicted gene, 25945 | Gm25945 |
| **-1.3** | 0.048767 | predicted gene, 26850 | Gm26850 |
| **-1.3** | 0.000632 | reproductive homeobox 3F | Rhox3f |
| **-1.31** | 0.012256 | microRNA mir-7091; mmu-mir-7091 | Mir7091; mmu-mir-7091 |
| **-1.31** | 0.023997 | microRNA mir-7212; mmu-mir-7212 | Mir7212; mmu-mir-7212 |
| **-1.31** | 0.00358 | nuclear encoded rRNA 5S 9 | n-R5s9 |
| **-1.31** | 0.009831 | olfactory receptor 113 | Olfr113 |
| **-1.31** | 0.009549 | predicted gene 11034 | Gm11034 |
| **-1.31** | 0.008327 | predicted gene 14700; novel pseudogene | Gm14700 |
| **-1.31** | 0.025205 | predicted gene 15963; Novel transcript | Gm15963 |
| **-1.31** | 0.003147 | predicted gene 20526; histocompatibility 2 (H2) pseudogene | Gm20526 |
| **-1.31** | 0.008747 | predicted gene 9874 | Gm9874 |
| **-1.31** | 0.045207 | predicted gene, 21746 | Gm21746 |
| **-1.31** | 0.004707 | predicted gene, 24786 | Gm24786 |
| **-1.31** | 0.001312 | predicted gene, 25076 | Gm25076 |
| **-1.31** | 0.000849 | predicted gene, 25260 | Gm25260 |
| **-1.31** | 0.014676 | predicted gene, 26800; novel transcript | Gm26800 |
| **-1.31** | 0.024332 | seven in absentia homolog 3 (Drosophila) | Siah3 |
| **-1.32** | 0.032194 | cytochrome b-245, beta polypeptide | Cybb |
| **-1.32** | 0.001322 | microRNA mir-7036; mmu-mir-7036 | Mir7036; mmu-mir-7036 |
| **-1.32** | 0.001926 | olfactory receptor 798 | Olfr798 |
| **-1.32** | 0.002456 | predicted gene 15336; novel transcript | Gm15336 |
| **-1.32** | 0.004183 | predicted gene, 25821 | Gm25821 |
| **-1.32** | 0.000093 | predicted gene, 26602 | Gm26602 |
| **-1.32** | 0.027358 | serine (or cysteine) peptidase inhibitor, clade A (alpha-1 antiproteinase, antitrypsin), member 7; serine (or cysteine) peptidase inhibitor, clade A (alpha-1 antipeptidase, antitrypsin), member 7 | Serpina7 |
| **-1.33** | 0.001485 | nuclear encoded rRNA 5S 179 | n-R5s179 |
| **-1.33** | 0.012364 | predicted gene, 22469 | Gm22469 |
| **-1.33** | 0.001104 | predicted gene, 24509 | Gm24509 |
| **-1.33** | 0.008592 | predicted gene, 25219 | Gm25219 |
| **-1.33** | 0.001104 | predicted gene, 25358 | Gm25358 |
| **-1.33** | 0.023163 | predicted gene, 25835 | Gm25835 |
| **-1.33** | 0.003446 | U3A small nuclear RNA | Rnu3a |
| **-1.33** | 0.000106 | yippee-like 2 (Drosophila) | Ypel2 |
| **-1.34** | 0.004808 | nuclear encoded rRNA 5S 155 | n-R5s155 |
| **-1.34** | 0.003396 | predicted gene, 24222 | Gm24222 |
| **-1.34** | 0.010784 | predicted gene, 24607 | Gm24607 |
| **-1.35** | 0.022552 | interferon inducible GTPase 1 | Iigp1 |
| **-1.35** | 0.001524 | microRNA 3074-2 | Mir3074-2 |
| **-1.35** | 0.000964 | microRNA mir-6337; mmu-mir-6337 | Mir6337; mmu-mir-6337 |
| **-1.35** | 0.000366 | nuclear encoded rRNA 5S 5 | n-R5s5 |
| **-1.35** | 0.021734 | nuclear encoded rRNA 5S 50 | n-R5s50 |
| **-1.35** | 0.023691 | predicted gene 12496; putative novel transcript | Gm12496 |
| **-1.35** | 0.002448 | predicted gene 5105; Novel protein (EG329763) | Gm5105 |
| **-1.35** | 0.013116 | predicted gene, 23322 | Gm23322 |
| **-1.35** | 0.003014 | predicted gene, 24767 | Gm24767 |
| **-1.36** | 0.028254 | microRNA mir-7064; mmu-mir-7064 | Mir7064; mmu-mir-7064 |
| **-1.36** | 0.001602 | nuclear encoded rRNA 5S 205 | n-R5s205 |
| **-1.36** | 0.004164 | predicted gene, 24547 | Gm24547 |
| **-1.37** | 0.00223 | microRNA mir-6384 | Mir6384 |
| **-1.37** | 0.001752 | predicted gene, 22507 | Gm22507 |
| **-1.37** | 0.003328 | predicted gene, 24976 | Gm24976 |
| **-1.37** | 0.027352 | predicted gene, 25885 | Gm25885 |
| **-1.37** | 0.000348 | vomeronasal 1 receptor 71 | Vmn1r71 |
| **-1.38** | 0.001766 | microRNA 290a; microRNA 290 | Mir290a; Mir290 |
| **-1.38** | 0.038025 | microRNA mir-7093; mmu-mir-7093 | Mir7093; mmu-mir-7093 |
| **-1.38** | 0.013929 | olfactory receptor 564; olfactory receptor 560 | Olfr564; Olfr560 |
| **-1.38** | 0.008534 | polymeric immunoglobulin receptor | Pigr |
| **-1.38** | 0.003473 | predicted gene, 23850 | Gm23850 |
| **-1.38** | 0.000202 | predicted gene, 24747 | Gm24747 |
| **-1.39** | 0.010999 | interleukin 1 receptor-like 2 | Il1rl2 |
| **-1.39** | 0.019202 | microRNA mir-7002; mmu-mir-7002 | Mir7002; mmu-mir-7002 |
| **-1.39** | 0.04147 | microRNA mir-8118; mmu-mir-8118 | Mir8118; mmu-mir-8118 |
| **-1.39** | 0.001331 | RIKEN cDNA C230021G24 gene | C230021G24Rik |
| **-1.39** | 0.00005 | T cell receptor alpha joining 2 | Traj2 |
| **-1.4** | 0.000773 | nuclear encoded rRNA 5S 89 | n-R5s89 |
| **-1.4** | 0.000361 | olfactory receptor 692 | Olfr692 |
| **-1.41** | 0.001354 | microRNA let7a-2 | Mirlet7a-2 |
| **-1.42** | 0.000242 | microRNA mir-6366; mmu-mir-6366 | Mir6366; mmu-mir-6366 |
| **-1.42** | 0.038378 | predicted gene 11172 | Gm11172 |
| **-1.42** | 0.002148 | predicted gene, 25558 | Gm25558 |
| **-1.43** | 0.000243 | predicted gene 10217 | Gm10217 |
| **-1.43** | 0.046396 | predicted gene, 19773 | Gm19773 |
| **-1.44** | 0.044644 | complement component 3a receptor 1 | C3ar1 |
| **-1.44** | 0.014594 | predicted gene, 24004 | Gm24004 |
| **-1.44** | 0.002954 | predicted gene, 26152 | Gm26152 |
| **-1.46** | 0.000798 | predicted gene, 24454 | Gm24454 |
| **-1.47** | 0.002439 | nuclear encoded rRNA 5S 161 | n-R5s161 |
| **-1.47** | 0.001295 | predicted gene, 22201 | Gm22201 |
| **-1.48** | 0.029403 | chemokine (C-X-C motif) ligand 14 | Cxcl14 |
| **-1.48** | 0.004142 | microRNA let7a-1 | Mirlet7a-1 |
| **-1.48** | 0.009138 | predicted gene, 17268 | Gm17268 |
| **-1.51** | 0.052315 | microRNA mir-6975; mmu-mir-6975 | Mir6975; mmu-mir-6975 |
| **-1.52** | 0.05096 | leucine-rich alpha-2-glycoprotein 1 | Lrg1 |
| **-1.57** | 0.002014 | nuclear encoded rRNA 5S 54 | n-R5s54 |
| **-1.63** | 0.012686 | microRNA 327 | Mir327 |
| **-1.63** | 0.012686 | microRNA 327 | Mir327 |
| **-1.64** | 0.000474 | aryl hydrocarbon receptor nuclear translocator-like | Arntl |
| **-1.7** | 0.000257 | cryptochrome 1 (photolyase-like) | Cry1 |
| **-1.7** | 0.018347 | microRNA mir-8115 | Mir8115 |
| **-1.72** | 0.000176 | RAR-related orphan receptor gamma | Rorc |
| **-1.73** | 0.043685 | microRNA 505 | Mir505 |
| **-1.74** | 0.04463 | hemoglobin, beta adult s chain; hemoglobin, beta adult major chain; hemoglobin, beta adult t chain; hemoglobin, beta adult minor chain | Hbb-bs; Hbb-b1; Hbb-bt; Hbb-b2 |
| **-1.85** | 0.015951 | C2 calcium-dependent domain containing 4B | C2cd4b |
| **-2.36** | 0.03653 | hemoglobin alpha, adult chain 2; hemoglobin alpha, adult chain 1 | Hba-a2; Hba-a1 |
| **-2.52** | 0.000389 | T cell receptor alpha joining 14 | Traj14 |
| **-2.66** | 0.038662 | hemoglobin alpha, adult chain 2; hemoglobin alpha, adult chain 1 | Hba-a2; Hba-a1 |
| **-2.73** | 0.03801 | hemoglobin, beta adult t chain; hemoglobin, beta adult minor chain; hemoglobin, beta adult s chain; hemoglobin, beta adult major chain | Hbb-bt; Hbb-b2; Hbb-bs; Hbb-b1 |
